# The Correlations of Anti-Mullerian Hormone, Follicle-Stimulating Hormone and Antral Follicle Count in Different Age Groups of Infertile Women

**DOI:** 10.22074/ijfs.2015.4179

**Published:** 2015-02-07

**Authors:** Ludmila Barbakadze, Jenara Kristesashvili, Natalia Khonelidze, Gia Tsagareishvili

**Affiliations:** 1 Department of Reproductology, Obstetrics and Gynecology, Medical Faculty, Ivane Javakhishvili Tbilisi State University, Tbilisi, Georgia; 2 Clinic for IVF and Human Reproductive Health, Tbilisi, Georgia

**Keywords:** Anti-Mullerian Hormone, Follicle Stimulating Hormone, Mullerian Inhibiting Substance

## Abstract

**Background:**

The objective of our study was to identify the correlations between the tests currently used in ovarian reserve assessment: anti-Mullerian hormone (AMH), follicle stimulating hormone (FSH) and antral follicle count (AFC) and to distinguish the most reliable markers for ovarian reserve in order to select an adequate strategy for the initial stages of infertility treatment.

**Materials and Methods:**

In this prospective study, 112 infertile women were assessed. Subjects were divided into three age groups: group I <35 years (n=39), group II 35-40 years (n=31), and group III 41-46 years (n=42). AMH, FSH and AFC were determined on days 2-3 of the patients’ menstrual cycles.

**Results:**

There was a significantly elevated negative correlation between age and AMH level (r_s_=-0.67, p<0.0001) and AFC (r_s_=-0.55, p<0.0001). We observed a significantly positive correlation between age and FSH (r_s_=0.38, p<0.0001). AMH negatively correlated with FSH (r_s_=-0.48, p<0.0001) and positively with AFC (r=-0.71, p=0.0001). There was a moderate negative relation between FSH and AFC (r=-0.41, p=0.0001) and moderate positive relation between age and FSH (r_s_=0.38, p<0.0001). The correlation analysis performed in separate groups showed that AMH and AFC showed a statistically significant positive correlation for group I (r=0.57, p<0.0001), group II (r=0.69, p<0.0001) and group III (r=0.47, p<0.002). A statistically significant correlation between FSH and AMH was detected only in groups I (r=-0.41, p<0.02) and II (r=-0.55, p<0.0001). A statistically significant correlation existed between FSH and AFC only in group III (r=-0.42, p<0.006), as well as between age and AFC only in group I (r=-0.35, p<0.03).

**Conclusion:**

Currently, AMH should be considered as the more reliable of the ovarian reserve assessments tests compared to FSH. There is a strong positive correlation between serum AMH level and AFC. The use of AMH combined with AFC may improve ovarian reserve evaluation.

## Introduction

In recent years assessment of ovarian reserve to determine the strategy for treatment of female infertility has become essential. Traditionally, age, follicle stimulating hormone (FSH), estradiol (E_2_) levels and antral follicle count (AFC) by ultrasound investigation at the early follicular phase have been used for evaluation of ovarian reserve. For years the levels of FSH and E_2_ were considered to be determining biochemical markers for assessment of low ovarian reserve. However, it has been found that the FSH level is above the norm only in cases when the ovary function is largely decreased (1). Later stage identification of the AFC is considered to be more reliable in assessment of the ovarian reserve. Follicle count can be determined easily with the help of high resolution sonographic systems (2-4). Although, there are well-known difficulties in obtaining correct AFC such as high inter-observer differences and anatomical variations. It has been suggested that AFC predicts poor response much better than basal FSH (3). Thus, by some investigators AFC is considered as the first choice test (2, 5).

Recently, identification of anti-Mullerian hormone (AMH) levels became important in assessment of ovarian reserve. AMH, also known as Mullerian-inhibiting substance, is a dimeric glycoprotein that belongs to the transforming growth factor – β family (6, 7). In reproductive-aged women AMH is expressed by small antral follicles. It is manifested by granulosa cells of the ovary (8). In the ovary AMH inhibits initial primordial follicle recruitment and decreases the sensitivity of pre-antral and small antral follicles to FSH (9). In comparison with other ovarian reserve assessment tests AMH is characterized by a number of advantages. AMH levels are stable throughout the menstrual cycle and therefore can be measured at any day of the cycle (8, 10). AMH levels are not affected by other hormonal variations, including the use of oral contraceptives (11). However, a recent study by Bentzen et al. has indicated that ovarian reserve markers are lower in women who use sex steroids for contraception. Thus, AMH concentration and AFC may not retain their accuracy as predictors of ovarian reserve in women who use hormonal contraception (12). AMH is not detected in women until puberty and reaches its highest levels at age 24.5 years (13). With increasing age, the number and quality of oocytes decline. Accordingly, the AMH level also declines and is lowest at menopause; later, it is not detected at all (11).

Recent studies have shown that follicular depletion doubles when the primordial follicle amount is approximately 25.000. Women reach this physiological condition at the ages of 37-38 years (13). This age is determined as critical, after which there is a sharp reduction in the ovarian reserve (14). This regularity is individual and changes in ovarian reserve can be associated not only with age. Thus, only a woman’s age is insufficient to determine ovarian reproductive potential. This creates the need for practical implementation of individual biological age-specific ovarian reserve determining tests, which can be highly reliable in assessment of a woman’s ovarian reserve and reproductive potential at the early stages of infertility. This should be especially taken into consideration in cases of infertile and sub-fertile women who need assisted reproductive technologies to achieve pregnancy. Recent studies have shown that AMH can be a good predictor of ovarian reserve and the success rates of *in vitro* fertilization (IVF) (15, 16). However, some studies have not found it to predict the power of pregnancy outcomes (17). According to data, even at low AMH levels, while it is considered as a pessimistic predictor in terms of reproductive potential, pregnancy can be still achieved (18).

As identification of AMH level for assessment of ovarian reserve is a recent method and obtained data are divergent, implementation of further studies and obtaining more materials in this field are viewed as justified and reasonable.

Hence, this study aimed to identify the correlations between current tests used in ovarian reserve assessment (AMH, FSH, AFC) in different age groups of infertile woman and distinguish the most reliable markers for ovarian reserve with the aim to select an adequate strategy for the initial stages of infertility treatment.

## Materials and Methods

This was a prospective study conducted on the basis of Tbilisi State University Medical Faculty Clinic and Clinic for IVF and Human Reproductive Health (Tbilisi, Georgia). Study population consisted of 112 infertile women who underwent infertility treatment from January, 2012 to February, 2013. Informed consent was obtained from all women and the study was approved by the Ethics Committee of the Clinic for IVF and Human Reproductive Health. Patients with previous ovarian surgery, polycystic ovarian syndrome (PCOS) and premature ovarian failure (POF) were excluded. We divided subjects into three age groups: group I <35 years (n=39), group II 35-40 years (n=31), and group III 41-46 years (n=42).

On 2-3 days of their spontaneous menstrual cycles, all patients underwent transvaginal scans conducted by the same investigator using a VOLUSON S6 (General Electric, USA, 2011y) with a 4-10 MHZ multi-frequency ultrasound probe. The numbers of antral follicles that measured 2-10 mm in size were counted in each ovary. The sum of both counts was the AFC. Levels of FSH and AMH were determined on the same days. Measurement of serum AMH level was performed using Gen II AMH Enzyme-linked Immunosorbent Assay (ELISA; Beckman Coulter, USA). FSH levels were assessed in plasma with the Enzyme-linked Fluorescence Assay (ELFA) on a mini VIDAS analyzer (BioMerieux SA, France).

### Statistical analysis

Statistical analysis was performed using SPSS (Statistical Package for the Social Sciences, version 21). Data were analysed by one-way ANOVA and the Kruskal Wallis test. Post-hoc comparisons were determined by the Bonferroni test, Spearman’s rho correlations and multiple linear regression analysis. The results in all the above mentioned procedures were accepted as statistically significant when the p-value was less than 5% (p<0.05).

## Results

**Fig 1:**
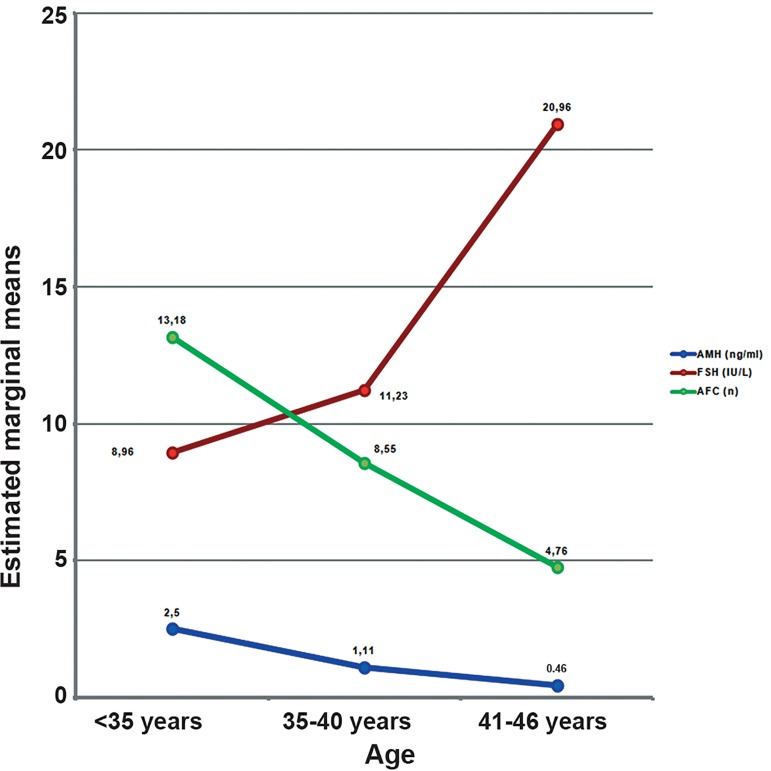
Mean AMH, FSH and AFC values in the three age groups. AMH; Anti-Mullerian hormone, FSH; Follicle stimulating hormone and AFC; Antral follicle count.

Distribution of the study population according to age groups was as follows: group I (35%), group II (28%) and group III (37%). Primary infertility was present in 59.3% (n=67) of patients. Figure 1 shows the values for AMH, FSH and AFC according to study group.

The three indicators of ovarian reserve significantly differed from each other in the different age groups (AMH: χ2=50.585, p=0.0001; FSH: χ2=15.566. p=0.0001; AFC: χ2 =34.386, p=0.0001). These indicators varied according to age.

Table 1 lists the differences between groups for the mean ± standard deviation AMH, FSH and AFC values. There were significantly higher AMH levels in group I compared with groups II and III. This value was also higher in group II compared to group III. AFC values were significantly higher in group I compared with group III, as well as in group II compared to group III. FSH levels were significantly higher only in group III compared to group I.

We examined the relationships between age and ovarian reserve indicators in all study groups. Age showed a significant negative correlation with AMH level (r_s_=-0.67, p<0.0001) and AFC (r_s_=-0.55, p<0.0001). There was a positive correlation between age and FSH (r_s_=0.38, p<0.0001). AMH showed a negative correlation FSH (r_s_=-0.48, p<0.0001) and a positive correlation with AFC (r=-0.71, p<0.0001). There was a significant negative correlation between FSH and AFC (r=-0.41, p<0.0001).

Correlation analysis performed in the separate groups showed that AMH and AFC levels had positive associations for group I (r=0.57, p<0.0001), group II (r=0.69, p<0.0001) and group III (r=0.47, p<0.002) which were significant. However only a significant correlation between FSH and AMH levels were detected in groups I (r=-0.41, p<0.02) and II (r=-0.55, p<0.0001). A statistically significant correlation between FSH and AFC was seen only in group III (r=-0.42, p<0.006), as well as between age and AFC in only group I (r=-0.35, p<0.03).

According to regression analysis, age only explained the variation of AMH in 22%, the variation of FSH in 14% and the variation of AFC in 27% of changes.

**Table 1:** Differences between age groups for mean FSH, AMH and AFC values


Indicators	<35 years	35-40 years	41-46 years

**Age (Y)**	28.75 ± 4.6	38.23 ± 1.72	43.29 ± 2.08
**AMH (ng/ml)**	2.5 ± 2.0	1.1 ± 1.11	0.46 ± 0.59
	p*<0.0001	p**<0.0001
		p***<0.0001
**FSH (IU/L)**	8.96 ± 3.46	11.23 ± 6.4	20.96 ± 19.84
	p*<0.623	p**<0.086
		p***<0.001
**Total AFC (n)**	13.18 ± 8.64	8.55 ± 4.5	4.76 ± 2.94
	p*<0.057	p**<0.0001
		p***<0.0001


Note: Values are represented with means and ± SD p*; Between groups I and II, p**; Between groups II and III, p*** Between groups I and III, AMH; Anti-Mullerian hormone, FSH; Follicle stimulating hormone and AFC; Antral follicle count.

## Discussion

The results obtained showed that ovarian reserve assessment tests in each age group reflected age-specific changes. The above mentioned trends were confirmed by other researchers (9, 19, 20). Of note, in the current study AMH values significantly differed in all three age groups, whereas AFC values were significantly higher in group I compared to group III and in group II compared to group III. FSH levels showed a significantly higher result only in group III compared to group I. Therefore AMH values reflected age-specific changes better than other indicators. Our findings were relative with the study of de Vet et al. (21) where early follicular phase hormone measurements at three-year intervals revealed that serum AMH levels declined significantly whereas serum levels of FSH and the number of antral follicles remained unchanged during this interval.

It is known that a woman’s age alone is insufficient to determine ovarian reproductive potential and this potential can be affected by various pathologies and iatrogenic conditions. Regression analysis of the current results have shown that changes in AMH, FSH and AFC levels were due to other known or unknown factors and therefore not only to age. According to our data the reduction in AMH and AFC levels by approximately one fourth was related to the increase in age. Approximately one sixth of the rate of change in FSH level could be attributed to age.

We examined relationships between age and ovarian reserve indicators in the whole study group and found that age had a highly significant negative correlation with AMH and AFC and a highly significant positive correlation with FSH level. However, the relation between the age and FSH was moderate (r_s_=0.38, p<0.0001). Thus, with age, AMH and AFC values strongly declined, whereas FSH levels moderately increased. The results reported by de Vet et al. also suggested that changes in serum AMH levels have been shown to occur relatively early in the sequence of events associated with ovarian aging (21). Elevated serum levels of FSH are not found until cycles become irregular (22). Therefore, a marker that already shows a considerable change when the cycle is still normal is a better indicator of women with declining fertility. The above mentioned results strongly suggest that serum AMH level can be used as a marker for ovarian aging.

In contrast to the total study group comparison, analysis within groups revealed quite interesting data in group I and the most sensitive age group II (35-40 years) where the correlation between serum FSH levels and AFC was not statistically significant. AMH and AFC in all three study groups showed a significant positive correlation. This positive correlation was confirmed by other researchers (23, 24).

There is no consensus on identification of the antral follicles , however several evidence based studies suggested to select the follicles as antral follicles based on a diameter measurement as 2 to 10 mm (4, 25). It has been reported that human antral follicles <6 mm express the most AMH; these levels decline with increasing antral follicle size (26). In a study by Goksedef et al. the best correlation was found between AMH levels and 5-6 mm antral follicles (27). In our study the number of 2-10 mm antral follicles was counted in the early follicular phase and a highly significant positive correlation between AMH and AFC values was found in all age groups. According to one recent study, a strong relationship between AMH and AFC was reported. This relationship was more significant than between the other typical biomarkers and AFC (23).

It is known that FSH level predicts poor responders during IVF (28). It has been identified in a meta-analysis that, possibly due to inter-cycle variability, basal FSH is not an accurate predictor of IVF outcome (29). AMH levels correlate with AFC and the number of retrieved oocytes that reflect the ovarian reserve. The vast majority of studies have also found that both AMH and AFC have similar values in predicting low response to ovarian stimulation (30, 31). A significant positive correlation between AMH levels and the quality (32) and quantity (33, 34) of oocytes has been found by several authors, although the value of AMH in predicting oocyte quality is controversial. Guerif et al. have reported that serum AMH was not predictive of oocyte quality during IVF (35). The presence of contradictory data necessitates carrying out further studies in this direction.

Considering the strong correlation between AMH and AFC, it is important to identify which of these two markers better predicts ovarian function, such as oocyte and embryo quality and IVF outcomes. Is it preferable to use these two indicators in combination or based only on AMH levels? Currently we are conducting research in this direction which will be the subject of a future publication.

## Conclusion

Currently, among ovarian reserve assesment tests used in modern practice, the AMH levels should be considered as more reliable. The results of the current study have shown that serum AMH levels are strongly related with AFC levels; this relationship is more significant than other ovarian reserve parameters. These results also indicate that the serum AMH measurement is a better predictor for the number of early antral follicles compared to conventional hormone measurements. Measuring AMH levels in combination with AFC may improve the assessment of ovarian reserve for evaluating fertility potential and monitoring infertility treatment.
